# Native and Heated Hydrolysates of Milk Proteins and Their Capacity to Inhibit Lipid Peroxidation in the Zebrafish Larvae Model

**DOI:** 10.3390/foods6090081

**Published:** 2017-09-14

**Authors:** Wilman Carrillo, Xavier Guzmán, Edgar Vilcacundo

**Affiliations:** Departamento de Investigación, Universidad Estatal de Bolívar, Av. Ernesto Che Guevara, Sector Alpachaca, Guaranda CP 020102, Ecuador; xavier.guzman@gmail.com (X.G.); geldphone@gmail.com (E.V.)

**Keywords:** milk proteins, caseins, whey proteins, inhibition of lipid peroxidation

## Abstract

Casein and whey proteins with and without heat treatment were obtained of whole milk and four commercial milks in Ecuador, and were hydrolyzed. Then, their capacity to inhibit the lipid peroxidation using the TBARS method was evaluated at concentrations of 0.02, 0.04, 0.2, and, 0.4 mg/mL. Native and heated hydrolysates of milk proteins present high inhibitions of lipid peroxidation with a dose dependent effect both in vivo and in vitro tests. Casein and whey proteins obtained from whole milk were the ones with the highest anti-oxidant activity in vitro and in vivo test. Native casein hydrolysate at 0.4 mg/mL present a value of 55.55% of inhibition of lipid peroxidation and heated casein hydrolysate at 0.4 mg/mL presents a value of 58.00% of inhibition of lipid peroxidation. Native whey protein at 0.4 mg/mL present a value of 34.84% of inhibition of lipid peroxidation, and heated whey protein at 0.4 mg/mL presents a value of 40.86% of inhibition of lipid peroxidation. Native and heated casein hydrolysates were more active than native and heated whey protein hydrolysates. Heat treatments have an effect of increasing the in vitro inhibition of lipid peroxidation of hydrolysates of milk protein. Casein and whey hydrolysates were able to inhibiting lipid peroxidation in the zebrafish larvae model. Native casein hydrolysate obtained of whole milk presents 48.35% of inhibition TBARS in vivo, this activity was higher in heated casein hydrolysate obtained of whole milk with a value of 56.28% of inhibition TBARS in vivo. Native whey protein hydrolysate obtained of whole milk presents 35.30% of inhibition TBARS, and heated whey protein hydrolysate obtained of whole milk was higher, with a value of 43.60% of inhibition TBARS in vivo.

## 1. Introduction

Cow milk and dairy products are food with a long tradition in human nutrition in the world. Cow milk is an excellent food as it contains the nutrients needed for growth and development of the calf, being a source of good lipids, proteins, amino acids, vitamins, and minerals. It contains immunoglobulins, hormones, growth factors, cytokines, nucleotides, peptides, polyamines, enzymes, and other bioactive peptides. Lipids in milk are emulsified in globules coated with membranes. Proteins are in colloidal dispersions as micelles. Casein micelles occur as colloidal complexes of protein and salts, primarily calcium [1,2]. 

Cow milk is an important source of bio compounds, especially bioactive peptides with different biological activities such as antimicrobial, antioxidant, antitumoral, antiviral, anti-inflammatory, antidiabetic, and antihypertensive activities. A considerable number of hydrolysates of caseins proteins and whey proteins to obtain bioactive peptides have been developed [3,4,5,6,7,8]. It has been reported that hydrolysates obtained with digestive enzymes from plants and animals origin and enzymes of bacteria sources. Peña-Ramos and Xiong have reported that soy protein isolate and its hydrolysates produce inhibition of lipid peroxidation using Tiobarbituric Acid Reactive Substances (TBARS) method by 28–65% [9]. They also reported whey protein isolate would be able to reduce TBARS reactive species. 

Carrillo et al. [10] reported the hen egg lysozyme and peptides of lysozyme with antioxidant activity, and with the capacity to inhibit lipid peroxidation (TBARS). They reported heated hen eggs white hydrolysates with a capacity to inhibit the lipid peroxidation in zebrafish larvae with cito-toxicity. Food proteins can be used to the inhibit lipid peroxidation of lipids by using both in vitro and vivo models. Moreover, soy and other food proteins, including egg white albumin [11], casein [12], and fish protein [13], were reported to contain antioxidant activities. The antioxidant activity was present in the enzymatic hydrolysates, being able to reduce the lipid and fatty acids peroxidation. Oxidative reactions in foods lead to the deterioration of quality attributes such as flavor, aroma, texture, and color. The main targets of oxidative reactions affecting food quality are lipids and proteins. With regards to lipids, these reactions typically involve the free radical pathways that result in the formation of lipid hydroperoxides, which are eventually decomposed to low molecular weight carbonyls in the presence of prooxidants. These carbonyls are associated with the rancid aroma and can interact with compounds such as proteins to alter functionality. 

The aim of this study was to potentiate the inhibition of lipid peroxidation in the in vitro and vivo zebrafish model with the heat treatment of cow milks.

## 2. Materials and Methods

### 2.1. Isolation of Caseins and Whey Proteins

Caseins were obtained by isoelectric precipitation from whole cow milk and four commercial milks in Ecuador by adding 2 M HCl (pH 4.6). Caseins were recovered by centrifuging at 4500 RPM for 15 min. The casein precipitate was washed three times with acidulated water (pH 4.6), and the remaining fat in the casein precipitate was removed by washing with dichloromethane acidulated water (1:1, *v*/*v*). Water was acidulated with 0.5 N HCl. The final casein precipitate was then lyophilized, and this product is referred to as total casein. The supernatant containing the whey proteins was removed, lyophilized, and stored at −20 °C [14]. 

### 2.2. Heat Treatment of Milk Proteins

1% (*w*/*v*) aqueous solution of casein and whey proteins were heated at 90 °C for 30 min, with constants stirring at 500 rmp. The heated solutions were centrifuged at 4500 rmp for 30 min, and the supernatant was lyophilized and stored at −20°C. 

### 2.3. Enzymatic Hydrolysis of Caseins and Whey Proteins

A 0.5% (*w*/*v*) aqueous solution of native and denatured casein or whey proteins was adjusted at pH 3.0 with 1 M HCl, and was added the porcine pepsin (3.7% *w*/*w* of substrate) for 3 h at 37 °C. The hydrolysates were centrifuged at 4500 rmp for 15 min. Inactivation of the pepsin enzyme was done by heating at 90 °C for 5 min. Finally, the supernatant was subjected to SDS-PAGE (sodium dodecyl sulphate-polyacrylamide gel electrophoresis) analysis. 

### 2.4. Sodium Dodecyl Sulphate-Polyacrylamide Gel Electrophoresis (SDS-PAGE)

SDS-PAGE electrophoresis of milk proteins and products of enzymatic hydrolysis of proteins were carried out according to the method proposed by Laemmli [15]. Runs were performed in Mini-protean (Bio-Rad, Hercules, CA, USA), SDS-PAGE system. Gels of 12 g acrylamide/100 mL of resolving gel, and 4 g acrylamide/100 mL of stacking gels were used. Relative molecular masses of protein were determined by comparison to the molecular weight marker with polypeptide SDS-PAGE standards (MW 10–250 and 6.5–200 kDa) (Bio-Rad, Hercules, CA, USA) and (14.4–97.0 kDa GE Healthcare Life Sciences, Uppsala, Sweden). Gels were fixed and stained with Coomassie Brilliant Blue G-250 (Sigma-Aldrich, St. Louis, MO, USA) for 16 h. 

### 2.5. Test of Toxicity in the Zebrafish Eggs Model

Zebrafish of the AB strain (wild-type, *wt*) embryos were obtained from natural spawning. Embryos were raised and fish were maintained as described by Westerfield [16]. After collection and disinfection, the eggs were placed in 24-well microplates with 1 mL of water, to study the in vivo toxicity of all samples with the zebrafish model. 

The assay was based on the Organisation for Economic Co-operation and Developmen (OECD) draft guideline on Fish Embryo Toxicity (FET) Test (OECD, 2013) [17], and is described in detail by Domingues et al. [18]. The Test Guideline is based on the chemical exposure of newly fertilized zebrafish eggs for up to 48 h, and is expected to reflect acute toxicity in fish in general. After 24 and 48 h exposure to milk proteins, four apical endpoints were recorded as indicators of acute lethality in fish: coagulation of fertilized eggs, lack of somite formation, lack of detachment of the tail-bud from the yolk sac, and lack of heart-beat. The eggs were considered dead when they exhibit at least one of the previous mentioned indicators. 

In the control wells, there should be less than 10% of the eggs with one of the mentioned indicators after 48 h [19]. Ten eggs per treatment (3 replicates) were selected and distributed in 24-well microplates. The test started with newly fertilized eggs exposed to the nominal concentrations of 0.625, 1.25, 2.5, 5.0, and 10 mg/mL of hydrolysates, and run for 2 days. Embryos were observed at 24 and 48 h under a stereomicroscope (magnification used in the stereomicroscope for observations was 40× using stereomicroscope Motic SMZ8 with camera Moticam 5 MP (Hong Kong, China). 

### 2.6. Test of Toxicity in the Zebrafish Larvae Model

The zebrafish colony was established in the laboratory, in an environmental growth or glass aquarium, provided with an internal filter and aerator activated carbon for water oxygenation. The population of animals was fed three times a day with food chips (Tetra S.A, Melle, Germany) for fish. Adult fish were kept on 16 h light and 8 h dark cycles. Embryos were obtained by photo-induced spawning over green plants and were cultured at 28 °C in fish tank water. Early larvae post-fertilization Zebrafish were maintained according to Kimmel et al. [20]. Larvae of 5 days post fecundation (DPF) were then incubated in 24-well plates, 30 larvae per well for each sample of milk hydrolysates at 0.4 mg/mL. The volume of fish tank water was 200 μL/well. The effect of each dilution placed in wells of 200 μL, with 30 larvae/well, was measured at 1, 24 and 36 h from incubation. After 48 h of treatment, the mortality as well as the morphologic changes were assessed. After their respective times, the percentage of larvae death in each dilution was determined. This percentage was plotted versus time. Groups from 30 larvae/well in aquarium water were used as controls. Stereoscopic microscope images were taken to obtain registration expressing the morphological effects on larvae anatomy, as compared to controls.

### 2.7. In Vitro Thiobarbituric Acid Reactive Substances (TBARS)

Caseins, whey protein, hydrolysates, and heated hydrolysates were used to evaluate the inhibition of lipid peroxidation. 0.5 g of olive oil was oxidized by heat at 65 °C for 8 days. Then, caseins, whey protein, hydrolysates, and heated hydrolysates were added to obtain concentrations of 0.02, 0.04, 0.1, and, 0.2 mg/mL of milk proteins, being incubated at 30 °C for 24 h. Butylhydroxytoluene (BHT) was used as positive control at concentrations of 0.02, 0.04, 0.1, and, 0.2 mg/mL of BHT. Olive oil without the anti-oxidant sample was used as a negative control. One milliliter of sample was mixed with one milliliter of the 1% thiobarbituric acid (TBA). The solution was heated at 95 °C during 1 h, and cooled down for 15 min. Then, absorbance of the final solution containing milk hydrolysates was measured at 532 nm using a spectrophotometer (Thermo Scientific Evolution 200, Madison, WI, USA). The decrease of absorbance indicates an increase of antioxidant activity. 

The values of antioxidant activity were expressed as the percentage of inhibition of lipid peroxidation as Equation (1):

%Inhibition of lipid peroxidation = As/Ab × 100(1)

where Ab is the absorbance of blank and As is the absorbance of the sample. 

### 2.8. In Vivo Thiobarbituric Acid Reactive Substances (TBARS) in Zebrafish Larvae Model

The thiobarbituric acid reactive species method was used, as described by Carrillo et al. [21]. Larvae were incubated in 24-well plates (30 larvae/well) with 2.0 mg/mL of milk digests. Groups of 30 larvae/well in aquarium water were used as controls. Lipid peroxidation was initiated by adding 1 mL of 500 μM H_2_O_2_, and incubated for 8 h at 28 °C. Then, H_2_O_2_ was removed with a micropipette and 500 μL of Tween 0.1% were added. Larvae were mixed and homogenized with a T25 Ultra turrax IKA (Wilmington, NC, USA). One milliliter of the 1% thiobarbituric acid (TBA) was added, and subsequently, the solution was heated at 95 °C for 1 h, and cooled down for 15 min. Then, the absorbance of the final solution containing zebrafish larvae and the protein milk digests was measured at 532 nm using by a spectrophotometer (Thermo Scientific Evolution 200, Madison, WI, USA). The decrease of absorbance indicates an increase of antioxidant activity. 

The values of antioxidant activity were expressed as the percentage of inhibition of lipid peroxidation in larvae homogenate as Equation (2):

%Inhibition of lipid peroxidation = (1 − (Ab − As)/Ab × 100)(2)

where Ab is the absorbance of blank, and As is the absorbance of the sample.

### 2.9. Statistical Analysis

Results are presented as means ± standard deviation from three replicates of each experiment. Differences between the mean values were determined by the analysis of variance (ANOVA). The post hoc analysis was performed by the Tuckey test. All tests were considered significant at *p* < 0.05. Statistical analysis was performed using the software package Prism 4 for Windows, version 6.0 (GraphPad Software Inc., CA, USA, www.graphpad.com).

## 3. Results

### 3.1. SDS-PAGE Electrophoresis Analysis

**Figure 1:**
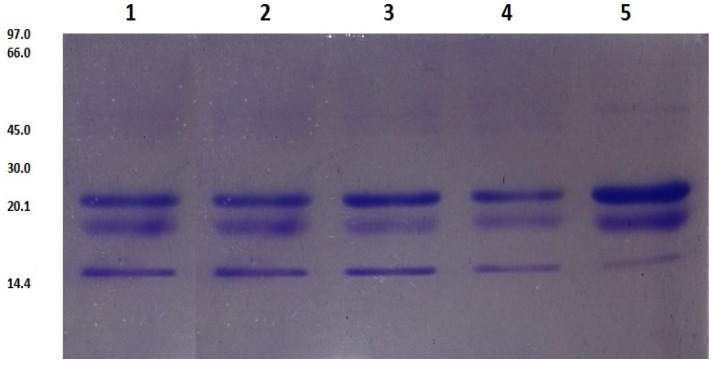
SDS-PAGE electrophoresis of caseins proteins with 2-mercaptoethanol. Lane 1: milk 1; lane 2: milk 2; lane 3: milk 3; lane 4: milk 4 and lane 5: whole cow milk.

**Figure 2:**
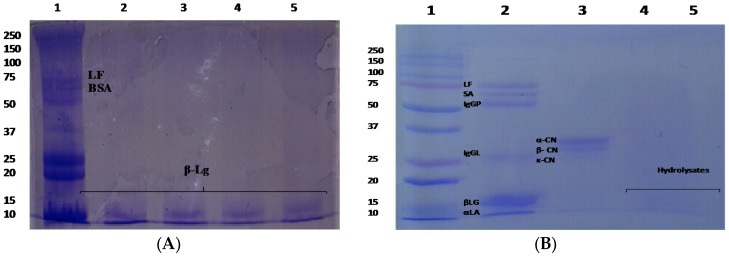
SDS-PAGE electrophoresis analysis of milk proteins with 2-mercaptoethanol. (**A**) Whey proteins. Lane 1: whole milk; lane 2: milk 1; lane 3: milk 2; lane 4: milk 3; lane 5: milk 4. (**B**) Hydrolysates. Lane 1: Standard protein; lane 2: whey protein; lane 3: casein proteins; lane 4: whey protein hydrolysate; and, lane 5: casein hydrolysate.

Caseins proteins were identified using SDS-PAGE electrophoresis. Figure 1 shows three bands in the isolate of casein that were identified: β CN; αS CN and *κ*-CN with weight between 18 to 25 kDa. These three bands were found in the whole milk and commercial milks tested. The bands identified, β CN; αS CN; and, *κ*-CN (Figure 1) were more intensive in whole milk. β-lactoglobulin, lactoferrin and albumin serum proteins were identified in the whole milk sample (Figure 2A). Whole casein and whey proteins were totally hydrolyzed with pepsin (Figure 2B). 

### 3.2. In Vitro Inhibition of Lipid Peroxidation (TBARS)

Lipid oxidation in foods results in an important number of secondary products. These products are mainly aldehydes, with the ability to produce oxidative damage. Longevity and high reactivity allows these molecules to act inside and outside the cells, interacting with biomolecules such as nucleic acids and proteins, often irreversibly damaging the delicate mechanisms involved in cell functionality. Malondialdehyde (MDA) is the principal and the most studied molecule of polyunsaturated fatty acid peroxidation. From the year 1960, different methods have been developed to analyse this molecule in order to quantify the level of oxidative stress in vivo and in vitro. Most assays to determine MDA have been developed on the basis of its derivatization with thiobarbituric acid (TBA). The condensation of these two molecules gives rise to a high absorptivity adduct, MDA-TBA, of pink color which can be easily assessed with a spectrophotometer [22,23].

In vitro antioxidant activity of caseins, whey proteins, and native and heated hydrolysates, at 0.02, 0.04, 0.2, and, 0.4 mg/mL were evaluated using the TBARS method. BHT was used as a positive control. BHT presents a high antioxidant activity with a range of 69.49% to 78.17% of inhibition of lipid peroxidation. BHT activity was dose dependent (Table 1 and Table 2). 

#### 3.2.1. Native Casein Hydrolysates

Native casein hydrolysates present a high antioxidant activity with a dose dependent effect. Native casein hydrolysate obtained from whole milk presents the highest activity with a range of 33.33% to 55.55% of in vitro inhibition of lipid peroxidation. The second highest active sample was milk 4 with a value between 25.42% to 40.67% of inhibition lipid peroxidation. The antioxidant activity presents a dose dependent effect (Table 1). Hu et al. [24] reported that milk protein could be used to inhibit the lipid oxidation in corn oil. The oxidative stability of the different protein-stabilized emulsions was in the order of casein > whey protein isolate > soy protein isolate, as determined by monitoring both the lipid hydroperoxide and headspace hexanal formation.

#### 3.2.2. Heated Caseins Hydrolysates

Heated casein hydrolysates obtained of whole milk was the sample with the highest antioxidant activity with a value range of 45.00% to 58.00% of inhibition of lipid peroxidation. Milk 4 presented an antioxidant activity with a value range of 31.63% to 46.70% of inhibition of lipid peroxidation. Heated hydrolysates were able to inhibit lipid peroxidation in a dose dependent manner. Heated casein hydrolysates were more active than the native casein hydrolysates. The heat treatment could increase their antioxidant capacity. This was true for all samples at all concentrations tested in this study (Table 1). 

#### 3.2.3. Native Whey Hydrolysates

Whole milk presented the highest value of inhibition of lipid peroxidation with a value range of 18.07% to 34.84% of inhibition of lipid peroxidation. The commercial milk presenting the highest result of antioxidant activity was the milk 4 sample, with a value of 14.31% to 30.88% of inhibition of lipid peroxidation. This antioxidant activity was dose dependant (Table 2). 

#### 3.2.4. Heated Whey Hydrolysates

Heated whey hydrolysates obtained of whole milk presented the better results of antioxidant activty, than native whey proteins, with a value of 26.93% to 40.86% of inhibition of lipid peroxidation. Milk 4 sample was the best commercial milk with antioxidant activity with a value of 20.90% to 34.08% of inhibition of lipid peroxidation. The heating treatment was able to increase the antioxidant activity of whey protein hydrolysates for all sample assays in this study (Table 2). 

When the antioxidant activity of casein hydrolysates was compared against whey proteins hydrolysates, it was observed that casein hydrolysates were more active than whey proteins hydrolysates. Heated casein hydrolysates were the sample with the highest antioxidant activity. Tong et al. [25], reported native and heated whey fractions from skim milk presenting a capacity to inhibit the peroxidation lipid in salmon oil. Whey protein and fractions were heated at 60, 70, 80 and 90°C for 15 min. The antioxidants values obtained to whole whey, HMW (high molecular whey) fraction, and LMW (less molecular whey) fraction whey inhibited oxidation 94.6, 86.3, and 58.0%, of inhibition of lipid peroxidation, respectively. They determined that the heat whey fractions increase the antioxidant activity of whey fractions. The best heat treatment was HMW heating at 80 °C for 15 min. Heat treatment produces the exposure of sulphydryls groups with antioxidant capacity. Moreover, Shon and Uhaque [26] reported inhibition of lipid peroxidation from native and heated sour whey protein in peanut oil, concluding that the native sour whey protein was more active than heated sour whey protein with a value of 55.7% of inhibition of lipid peroxidation in peanut oil, whereas heated sour whey protein presented a value of 52.1% of inhibition of lipid peroxidation in peanut oil. Heat treatment of whey proteins produced an increase of their antioxidant activity, and this study the hydrolysates from milk increased their capacity to inhibite of lipid peroxidation in vitro and in vivo model. 

Different scientific studies have reported food proteins with a high capacity to inhibit lipid oxidation. These food proteins are obtained of animal and vegetal sources such as milk, egg, soybean, and blood plasma. Porcine blood plasma (2.5%) contains antioxidant proteins such as serum albumin and transferrin, and can retard the formation of thiobarbituric acid reactive substances (TBARS) in both salted ground pork [27] and cooked ground beef. Whey protein concentrate is antioxidative in cooked beef [28], and whey and soy proteins inhibit lipid oxidation in cooked pork patties containing 2% protein [9], with soy protein isolate being more effective than whey proteins. Whey proteins have also been found to inhibit lipid oxidation in oil-in-water emulsions [29,30,31,32,33]. Park et al. [34], found that the soy protein inhibited the oxidation of ethyl esters of eicosapentaenoic acid dried in a maltodextrin-stabilized, freeze-dried emulsion powder system.

### 3.3 In Vivo Inhibition of Lipid Peroxidation (TBARS) in the Zebrafish Larvae Model 

**Figure 3:**
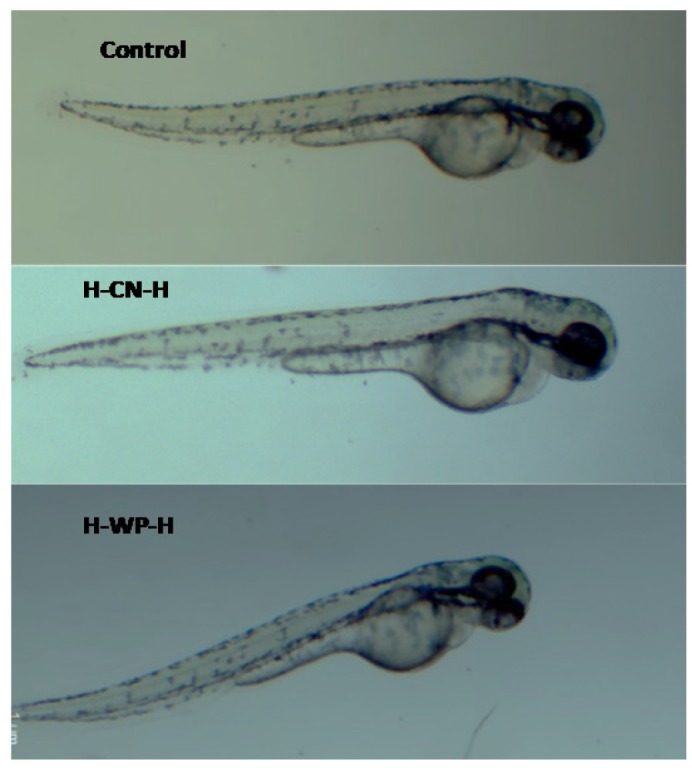
Test of toxicity in zebrafish larvae model incubated with milk proteins for 48 h at 2.5 mg/mL. Control (distillated water), H-CN-H (Hetaed caseines hydrolysate) and H-WP-H (Heated whey protein hydrolysate).

**Figure 4:**
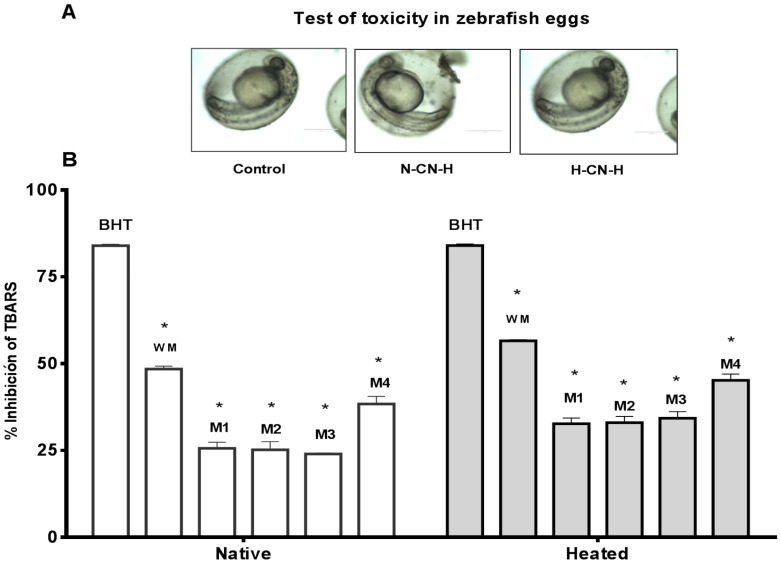
(**A**) Antioxidant activity of casein hydrolysates at concentration of 0.4 mg/mL. Tiobarbituric Acid Reactive Substances (TBARS) in zebrafish larvae model BHT (positive control). Native = native casein hydrolysate, Heated = heated casein hydrolysate. WM = whole milk, M1 = milk 1, M2 = milk 2, M3 = milk 3, M4 = milk 4. Results represent the average of two determinations ± SD (*n* = 30). * Denotes *p* < 0.05 as compared to positive control (two-way ANOVA/Tukey); (**B**) Test of toxicity in zebrafish eggs of N-CN-H (native caseins hydrolysate) and H-CN-H (heated caseins hydrolysate).

**Figure 5:**
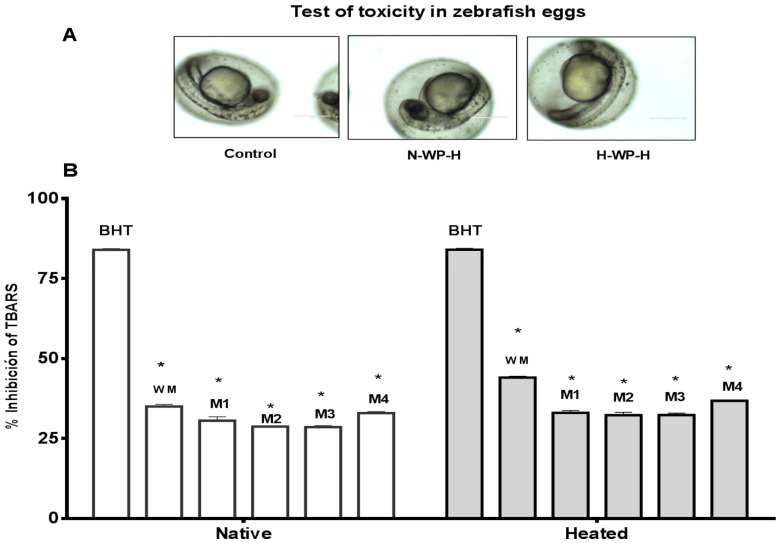
(**A**) Antioxidant activity of whey protein hydrolysate at concentration of 0.4 mg/mL. TBARS in zebrafish larvae model BHT (positive control). Native = native casein hydrolysate, Heated = heated casein hydrolysate. WM = whole milk, M1 = milk 1, M2 = milk 2, M3 = milk 3, M4 = milk 4. Results represent the average of two determinations ± SD (*n* = 30). * Denotes *p* < 0.05 as compared to positive control (two-way ANOVA/Tukey); (**B**) Test of toxicity in zebrafish eggs of N-WP-H (native whey protein hydrolysate) and H-WP-H (heated whey protein hydrolysate).

Cytotoxicity of native and heated hydrolysates from caseins and whey was evaluated using the in vivo zebrafish larvae model. After 48 h of incubation of zebrafish larvae with the hydrolysates, the zebrafish larvae exhibited the same morphology than the control (non-treated) (Figure 3). These results indicate that the hydrolysates from milk proteins showed an absence of cytotoxic macroscopic effects. Morevoer, the cytotoxicity of native and heated hydrolysates from caseins, and whey was evaluated using the in vivo zebrafish eggs model. After 48 h of exposure of zebrafish eggs with the samples showed no cytotoxic effects in the morpholgy of zebrafish embrions. At 72 h, all of the eggs hatched and zebrafish larave presented normal movement (Figure 4A and Figure 5A). 

Antioxidant activity of caseins, whey proteins and native and heated hydrolysates at 0.4 mg/mL were evaluated using the in vivo TBARS method in zebrafish larvae model. BHT was used as positive control at a concentration of 0.4 mg/mL. This concentration presented high antioxidant activity with a value of 84.250% of inhibition of lipid peroxidation (Figure 4B). 

#### 3.3.1. Native Casein Hydrolysates

Native casein hydrolysates present a high inhibition of lipid peroxidation in the zebrafish larvae model at a concentration of 0.4 mg/mL after 48 h of incubation in the sample. Native casein hydrolysate obtained of whole milk was the sample with the highest antioxidant activity, with a value of 48.35% of inhibition of lipid peroxidation. The commercial milk with the best activity was milk 4 with a value of 38.50%. This result shows a correlation with the results obtained of native casein hydrolysate when evaluated in vitro using the TBARS method (Figure 4B). 

#### 3.3.2. Heated Caseins Hydrolysates

The whole milk sample was again the best sample, with a value of 56.28% of inhibition of lipid peroxidation using the zebrafish larvae method. The second sample with the highest activity was milk 4, with a value of 43.50% of inhibition of lipid peroxidation in zebrafish larvae. Heat treatment increased the antioxidant activity of casein hydrolysates as compared to native casein hydrolysates, although these values were lower than the in vitro evaluation using the TBARS model (Figure 4B). 

#### 3.3.3. Native Whey Hydrolysates

Native whey hydrolysates were used to evaluate the inhibition of lipid peroxidation in the zebrafish larvae model at a concentration of 0.4 mg/mL after 48 h of incubation with the hydrolysates. Native whey was active to inhibit the lipid peroxidation in the zebrafish larvae model. Whole milk presented a higher activity with a value of 35.30% of inhibition of lipid peroxidation in the zebrafish larvae model, followed by milk 4 with a value of 32.30% of inhibition of lipid peroxidation in the zebrafish larvae model (Figure 5B). 

#### 3.3.4. Heated Whey Hydrolysates

Heated whey hydrolysates were used to evaluate the inhibition of lipid peroxidation in the zebrafish larvae model at a concentration of 0.4 mg/mL after 48 h of incubation with the hydrolysates. Heated whey hydrolysates were able to inhibit the lipid peroxidation in the zebrafish larvae model. As in previous cases, whole milk was the sample with the highest activity, with a value of 43.60% of inhibition using the TBARS method, followed by milk 4 with a value of 36.70% of inhibition of TBARS in zebrafish larvae (Figure 5B). Likewise, the thermal treatment increased the activity of the samples. The results of antioxidant activity in the in vitro model were higher than the values obtained with the in vivo zebrafish larvae model. For example, native caseins hydrolysates at 0.4 g/mL present a value of 55.55% of inhibition of lipid peroxidation in vitro, while the native caseins hydrolysates at 0.4 mg/mL present a value of 48.35% of inhibition lipid peroxidation. These results have statistical differences. In the in vivo model, the response depends on the cellular metabolism of the larvae. The mechanims implicated can be complex. 

## 4. Conclusions

Casein hydrolysate and whey proteins hydrolysates presented a high antioxidant activity. Native casein hydrolysates obtained of whole cow milk and commercial Milk 4 at 0.4 mg/mL presented a value of 55.55% and 40.67% of inhibition of lipid peroxidation. Heat treatment increased the antioxidant activity of casein hydrolysates and whey proteins hydrolysates. Heat casein hydrolysates at 0.4 mg/mL presented the highest activity with a value of 58.00% and 46.70% of the inhibition of lipid peroxidation for the whole milk and milk 4, respectively. Milk proteins are then effective to inhibit the lipid peroxidation. These proteins can be used as natural antioxidants without toxicity.

## Figures and Tables

**Table 1:** Antioxidant activity of native and heated casein hydrolysates obtained of whole cow milk and four commercial cow milk from Ecuador.

Treatment	Sample	% Inhibition of Lipid Peroxidation (TBARS) of Caseins Hydrolysates (mg/mL)			
		0.02	0.04	0.2	0.4
Hydrolysis	BHT	69.49 ± 0.26 ^a^	74.57 ± 0.46 ^a^	77.96 ± 0.70 ^a^	78.71 ± 0.26 ^a^
	Whole milk	33.33 ± 2.39 ^b^	44.25 ± 0.26 ^b^	51.60 ± 1.33 ^b^	55.55 ± 0.26 ^b^
	Milk 1	14.87 ± 0.96 ^c^	21.46 ± 0.46 ^c^	26.55 ± 0.92 ^c^	30.88 ± 0.96 ^c^
	Milk 2	18.83 ± 0.70 ^d^	24.48 ± 1.48 ^d^	29.37 ± 1.66 ^d^	36.72 ± 1.22 ^d^
	Milk 3	19.77 ± 0.70 ^e^	25.98 ± 1.66 ^e^	30.69 ± 0.70 ^e^	37.85 ± 0.46 ^e^
	Milk 4	25.42 ± 0.46 ^f^	32.20 ± 0.46 ^f^	35.97 ± 0.70 ^f^	40.67 ± 1.84 ^f^
Heat + hydrolysis	Whole milk	45.00 ± 0.53 ^a^	50.09 ± 0.53 ^a^	53.48 ± 0.53 ^a^	58.00 ± 0.26 ^a^
	Milk 1	19.20 ± 0.92 ^b^	25.80 ± 0.26 ^b^	32.20 ± 0.46 ^b^	37.66 ± 0.26 ^b^
	Milk 2	24.48 ± 0.70 ^c^	30.88 ± 0.26 ^c^	38.04 ± 1.16 ^c^	42.37 ± 0.46 ^c^
	Milk 3	25.42 ± 0.46 ^d^	31.26 ± 0.70 ^d^	39.73 ± 0.70 ^d^	46.51 ± 0.96 ^d^
	Milk 4	31.63 ± 0.46 ^e^	35.02 ± 0.92 ^e^	42.75 ± 0.26 ^e^	46.70 ± 1.86 ^e^

Data are expressed as the mean ± standard deviation (*n* = 3). Values in the same column having different letters differ significantly (*p* < 0.05). ANOVA and Tukey’s test. BHT: Butylhydroxytoluene.

**Table 2:** Antioxidant activity of native and heated whey proteins hydrolysates obtained of whole cow milk and four commercial cow milks from Ecuador.

Treatment	Sample	% Inhibition of Lipid Peroxidation (TBARS) of Whey Protein Hydrolysates (mg/mL)			
		0.02	0.04	0.2	0.4
Hydrolysis	BHT	69.49 ± 0.26 ^a^	74.57 ± 0.46 ^a^	77.96 ± 0.70 ^a^	78.71 ± 0.26 ^a^
	Whole milk	18.07 ± 0.92 ^b^	25.23 ± 0.70 ^b^	28.43 ± 1.48 ^b^	34.84 ± 0.70 ^b^
	Milk 1	10.73 ± 0.46 ^c^	18.07 ± 0.46 ^c^	21.09 ± 0.96 ^c^	29.19 ± 5.34 ^c^
	Milk 2	12.42 ± 1.22 ^d^	19.77 ± 0.46 ^d^	22.78 ± 0.70 ^d^	30.50 ± 0.46 ^d^
	Milk 3	13.37 ± 1.06 ^e^	21.09 ± 0.96 ^e^	25.23 ± 0.70 ^e^	27.49 ± 0.26 ^e^
	Milk 4	14.31 ± 0.26 ^f^	19.77 ± 0.46 ^d^	25.23 ± 0.70 ^e^	30.88 ± 0.26 ^f^
Heat + hydrolysis	Whole milk	26.93 ± 0.70 ^a^	29.00 ± 0.70 ^a^	33.52 ± 0.70 ^a^	40.86 ± 0.26 ^a^
	Milk 1	14.12 ± 0.46 ^b^	19.20 ± 1.38 ^b^	26.36 ± 1.48 ^b^	31.63 ± 0.79 ^b^
	Milk 2	17.51 ± 0.46 ^c^	21.46 ± 0.46 ^c^	26.36 ± 0.96 ^c^	34.27 ± 1.92 ^c^
	Milk 3	18.83 ± 0.70 ^d^	23.16 ± 2.01 ^d^	28.43 ± 0.96 ^d^	34.08 ± 0.70 ^c^
	Milk 4	20.90 ± 1.22 ^e^	25.42 ± 0.46 ^e^	29.56 ± 1.16 ^e^	34.08 ± 0.70 ^c^

Data are expressed as the mean ± standard deviation (*n* = 3). Values in the same column having different letters differ significantly (*p* < 0.05). ANOVA and Tukey’s test.

## References

[1] Pardo M.F., Natalucci C.L. (2002). Electrophoretic analysis (Tricine-SDS-PAGE) of bovine caseins. Acta Farm. Bonaer..

[2] Jovanovic S., Barac M., Macej O., Vucic T., Lacnjevac C. (2007). SDS-PAGE analysis of soluble proteins in reconstituted milk exposed to different heat treatments. Sensors.

[3] Meisel H. (2004). Multifunctional peptides encrypted in milk proteins. Biofactors.

[4] You S.J., Udenigwe C.C., Aluko R.E., Wu J. (2010). Multifunctional peptides from egg white lysozyme. Food Res. Int..

[5] Lönnerdal B. (2016). Human Milk: Bioactive Proteins/Peptides and Functional Properties. Protein in Neonatal and Infant Nutrition: Recent Updates.

[6] Abdel-Hamid M., Otte J., De Gobba C., Osman A., Hamad E. (2017). Angiotensin I-converting enzyme inhibitory activity and antioxidant capacity of bioactive peptides derived from enzymatic hydrolysis of buffalo milk proteins. Int. Dairy J..

[7] Hernández-Ledesma B., Dávalos A., Bartolomé B., Amigo L. (2005). Prepa-ration of antioxidant enzymatic hydrolysates from alpha-lactalbumin and beta-lactoglobulin. Identification of active peptides by HPLC-MS/MS. J. Agric. Food Chem..

[8] Chen H.M., Muramoto K., Yamauchi F. (1995). Structural Analysis of Antioxidative Peptides from Soybean β-Conglycinin. J. Agric. Food Chem..

[9] Peña-Ramos E.A., Xiong Y.L. (2003). Whey and soy protein hydrolysates inhibit lipid oxidation in cooked pork patties. Meat Sci..

[10] Carrillo W., Gómez-Ruiz J.A., Miralles B., Ramos M., Barrio D., Recio I. (2016). Identification of antioxidants peptides of hen egg White Lysozyme and evaluation of inhibition of lipid peroxidation and cytotoxicity in the zebrafish model. Eur. Food Res. Technol..

[11] Tsuge N., Eikawa Y., Nomura Y., Yamamoto M., Sugisawa K. (1991). Antioxidative activity of peptides prepared by enzymatic hydrolysis of egg-white albumin. Nippon Nogeikagaku Kaishi.

[12] Sakanaka S., Tachibana Y., Ishihara N., Juneja L.R. (2005). Antioxidant properties of casein calcium peptides and their effects on lipid oxidation in beef homogenates. J. Agric. Food Chem..

[13] Amarowicz R., Shahidi F. (1997). Antioxidant activity of peptide fractions of capelin protein hydrolysates. Food Chem..

[14] Rodríguez Saint-Jean S., las Heras A., Carrillo W., Recio I., Ortiz-Delgado J.B., Ramos M., Pérez-Prieto S.I. (2013). Antiviral activity of casein and αs2 casein hydrolysates against the infectious haematopoietic necrosis virus, a rhabdovirus from salmonid fish. J. Fish Dis..

[15] Laemmli U.K. (1970). Cleavage of structural proteins during the assembly of bacteriophage T4. Nature.

[16] Westerfield M. (1995). The Zebrafish Book. A Guide for the Laboratory Use of Zebrafish (Danio Rerio).

[17] OECD (2013). Test No. 236: Fish Embryo Acute Toxicity (FET) Test.

[18] Domingues I., Oliveira R., Lourenco J., Grisolia C.K., Mendo S., Soares A.M. (2010). Biomarkers as a tool to assess effects of chromium (VI): Comparison of responses in zebrafish early life stages and adults. Comp. Biochem. Physiol. C Toxicol. Pharmacol..

[19] Nagel R. (2002). DarT: The embryo test with the Zebrafish Danio rerio a general model in ecotoxicology and toxicology. Altex.

[20] Kimmel C.B., Ballard W.W., Kimmel S.R., Ullmann B., Schilling T.F. (1995). Stages of embryonic development of the zebrafish. Dev. Dyn..

[21] Carrillo W., Tubón J., Vilcacundo R. (2016). Isolation of hen egg white lysozyme by cation exchange chromatography, analysis of its digestibility and evaluation of the inhibition lipid peroxidation in the zebrafish model. Asian J. Pharm. Clin. Res..

[22] Wu S.J., Ng L.T. (2008). Antioxidant and free radical scavenging activities of wild bitter melon (*Monordica charantia* Linn. Var. *abbreviate* Ser.) in Taiwan. LWT Food Sci. Technol..

[23] VanderVeen L.A., Hashim M.F., Shyr Y., Marnett L.J. (2003). Induction of frameshift and base pair substitution mutations by the major DNA adduct of the endogenous carcinogen malondialdehyde. Proc. Natl. Acad. Sci. USA.

[24] Hu M., McClements D.J., Decker E.A. (2003). Lipid oxidation in corn oil-in-water emulsions stabilized by casein, whey protein isolate, and soy protein isolate. J. Agric. Food Chem..

[25] Tong L.M., Sasaki S., McClements D.J., Decker E.A. (2000). Antioxidant activity of whey in a salmon oil emulsion. J. Food Sci..

[26] Shon J., Haque Z.U. (2007). Antioxidative ability of native and thermized sour whey in oxidation-catalysed model systems. Int. J. Dairy Technol..

[27] Faraji H., Decker E.A., Aaron D.K. (1991). Suppression of lipid oxidation in phosphatidylcholine liposomes and ground pork by spray-dried porcine plasma. J. Agric. Food Chem..

[28] Shantha N.C., Decker E.A. (1994). Rapid, sensitive, iron-based spectrophotometric methods for determination of peroxide values of food lipids. J. AOAC Int..

[29] Taylor M.J., Richardson T. (1980). Antioxidant activity of skim milk: Effect of heat and resultant sulfhydryl groups. J. Dairy Sci..

[30] Allen J.C., Wrieden W.L. (1982). Influence of milk proteins on lipid oxidation in aqueous emulsion. I. Casein, whey protein, and a-lactalbumin. J. Dairy Res..

[31] Allen J.C., Wrieden W.L. (1982). Influence of milk proteins on lipid oxidation in aqueous emulsion. II. Lactoperoxidase, lactoferrin, superoxide dismutase, and xanthine oxidase. J. Dairy Res..

[32] Elias R.J., McClements D.J., Decker E.A. (2005). Antioxidant activity of cysteine, tryptophan, and methionine residues in continuous phase beta-lactoglobulin in oil-in-water emulsions. J. Agric. Food Chem..

[33] Park E.Y., Murakami H., Mori T., Matsumura Y. (2005). Effects of protein and peptide addition on lipid oxidation in powder model system. J. Agric. Food Chem..

[34] Donnelly J.L., Decker E.A., McClements D.J. (1998). Iron-catalyzed oxidation of menhaden oil as affected by emulsifiers. J. Food Sci..

